# The effects of special educational needs and socioeconomic status on teachers' and parents' judgements of pupils' cognitive abilities

**DOI:** 10.1111/bjep.12719

**Published:** 2024-10-03

**Authors:** Kim Smeets, Ellen Rohaan, Sanne van der Ven, Anouke Bakx

**Affiliations:** ^1^ Developmental Psychology Tilburg University Tilburg the Netherlands; ^2^ Stichting BOOM Oisterwijk the Netherlands; ^3^ Fontys School for Child Studies and Education, Fontys University of Applied Sciences Eindhoven the Netherlands; ^4^ Behavioural Science Institute, Radboud University Nijmegen the Netherlands; ^5^ Fontys School for Child Studies and Education, Fontys University of Applied Sciences Tilburg the Netherlands; ^6^ PPF Centrum voor Hoog OntwikkelingsPotentieel Vught the Netherlands

**Keywords:** cognitive abilities, giftedness, multilevel analysis, parent judgements, socioeconomic status, special educational needs, teacher judgements

## Abstract

**Background:**

Teachers' and parents' judgements of pupils' cognitive abilities influence pupils' daily learning opportunities and experiences, as these judgements affect the difficulty level of materials and instruction that teachers and parents provide. Over time, these judgements thus significantly shape educational success. However, pupils' characteristics, such as special educational needs (SEN), giftedness and socioeconomic status (SES) can influence and bias judgement accuracy.

**Aims:**

The present study aimed to investigate the relation between pupils' cognitive abilities and their teachers' and parents' judgements of these abilities, and potential bias in these judgements related to SEN, giftedness, and SES.

**Sample:**

The sample consisted of 1073 primary school pupils from grades 4–6 from 77 classes in 16 schools, and their teachers and parents.

**Methods:**

Teachers and parents rated their pupils' cognitive abilities. Pupils completed the COVAT‐3, a cognitive ability test.

**Results:**

Multilevel analyses revealed that parent judgements were significantly higher than teacher judgements, but both informants' judgements were equally strong related to the cognitive ability scores. When controlling for pupils' assessed cognitive abilities, the results revealed small judgement biases: negative for SEN, positive for giftedness, and finally positive for high SES, but only in teachers.

**Conclusions:**

Overall, the results indicated that teachers and parents can judge their pupils abilities to a moderate degree, but they also hold judgement biases related to SEN, giftedness and SES. As these biases can affect pupils' opportunities, it is important to increase teachers' and parents' awareness.

## BACKGROUND

Accurate teacher judgements are at the basis of teachers' professional competencies (Baumert & Kunter, 2011). These judgements play an essential role in pupils' personal growth and academic outcomes (Machts et al., 2016; Wang et al., 2018). They guide teachers' decisions about instruction (Parsons et al., 2018), working materials, grades, and ability grouping (Machts et al., 2016; Südkamp et al., 2012). In addition, teachers' judgements also (subconsciously) influence their behaviour towards pupils (Gentrup et al., 2020; Rubie‐Davies, 2007).

Teachers should adopt a holistic perspective to accurately assess pupils' needs, reflecting teacher sensitivity (Van Manen, 2008). A comprehensive approach considers various facets of the pupil and helps developing a nuanced understanding. However, teachers often primarily focus on academic abilities and achievement (Deunk et al., 2018). Cognitive abilities are the foundation of academic capabilities and achievements (Deary et al., 2007; Jabůrek et al., 2022). Yet the relation between academic achievement and cognitive abilities is limited, sharing only 29% variance (Roth et al., 2015). For example, pupils may underachieve, hiding their full potential (White et al., 2018), which is frequently overlooked by teachers (Rost & Hanses, 1998). Teachers should thus possess the ability to look beyond mere achievement and consider other factors in forming their judgements. This study focuses on the judgements of cognitive abilities, because these are crucial aspects of pupil potential (Gnas et al., 2022).

Besides teachers, parents play a crucial role in pupils' lives (Benner et al., 2021). Parental judgements about their children's cognitive abilities and, related to this, their educational expectations, influence children's grades, (aspired) educational attainment, attribution style, self‐perception of competencies (Pinquart & Ebeling, 2020; Pomerantz & Dong, 2006; Sorhagen, 2014), motivation, and academic and social resilience (Yamamoto & Holloway, 2010). Both teachers' and parents' judgements of pupils' cognitive abilities could lead to a self‐fulfilling prophecy (Gentrup et al., 2020; Pomerantz & Dong, 2006).

Studies investigating the relation between teachers' and parents' judgements of pupils' cognitive abilities and pupils' measured cognitive abilities, generally find moderate relationships (e.g., Chamorro‐Premuzic et al., 2009; Machts et al., 2016). Teachers' judgements often align stronger with assessed cognitive abilities than those of parents (Chamorro‐Premuzic et al., 2009). However, when teachers' and parents' judgements are aligned, it can enhance pupils' academic achievement (Peet et al., 1997). Moreover, the relatively accurate judgements do not hold for all groups of pupils, as pupils' (demographic) characteristics play a role in judgement accuracy (e.g., Wang et al., 2018; Yamamoto & Holloway, 2010); Pupils' special educational need status (SEN), giftedness, and socioeconomic status (SES) influence these judgements (e.g., Cosgrove et al., 2014; Jenkins & Demaray, 2016).

Considering that judgements influence daily practices such as tailored instruction, selection of materials, and shaping of self‐perception, with consequential long‐term effects on pupils' educational success, their social and economic opportunities as well as their future health (Fischbach et al., 2013; Gentrup et al., 2020; Urhahne, 2015), it is important that these judgements are accurate and free of bias (Wollschläger, 2016). Particularly for pupils who are underestimated (Gentrup et al., 2020), ensuring accurate and unbiased judgements allows for better support aligned with each pupil's potential. The present study therefore aimed to gain insight into the relation between pupils' cognitive abilities and their teachers' and parents' judgements of these abilities. In addition, this study aimed to investigate potential sources of systematic bias in these judgements caused by SEN, giftedness, and SES.

### Judgement of cognitive abilities

Research on teacher and parent judgements has mainly focused on academic achievement (e.g., Urhahne & Wijnia, 2021). Nevertheless, pupils' potential, encompassing cognitive abilities (Deary et al., 2007), does not consistently align with their academic accomplishments (White et al., 2018). Particularly during primary school years, a child's potential unfolds dynamically (Baudson et al., 2016), and cognitive capacities remain highly adaptable (Rose & Fischer, 2011). For instance, the meta‐analysis of Roth et al. (2015) found a weaker correlation between intelligence and school grades during primary education (ρ = .45) compared to secondary education (ρ = .54 to .58). Thus, examining assessments of pupils' cognitive abilities also offers valuable insights.

Previous studies exploring cognitive ability judgements have often treated intelligence or cognitive ability as singular constructs (Machts et al., 2016). However, the concept of a single underlying g‐factor for general cognitive ability is debated (Van der Maas et al., 2006). Intelligence comprises multiple cognitive abilities (McGrew et al., 2023). Proficiency in cognitive abilities manifests diversely among individuals, with each individual exhibiting strengths and weaknesses across various cognitive abilities. Approaching cognitive abilities as unidimensional fails to capture this complexity (Schneider & Newman, 2015). Carrol (1993, p. 10) defined cognitive abilities as the abilities that relate to ‘any task in which correct and appropriate processing of mental information is critical to successful performance’. The Cattell–Horn–Carroll theory of cognitive abilities (CHC theory) is the most widely recognized and empirically tested psychometric taxonomy of cognitive abilities (McGrew et al., 2023). The theory divides intelligence into 10 broad cognitive abilities (McGrew, 2009): fluid reasoning (reasoning), comprehension knowledge (comprehension), short‐term memory (STM), visual processing, auditory processing, long‐term storage and retrieval (LTM), reaction and decision speed, reading and writing, quantitative knowledge, and processing speed. In the present study, we refer to the CHC theory when addressing cognitive abilities.

Recognizing the diversity of cognitive abilities and acknowledging that individuals may excel or struggle in the distinct cognitive abilities, this study includes each cognitive ability independently.

### Defining teacher and parent judgements

Teacher (and also parent) judgement accuracy is the ability to adequately assess pupils' characteristics (Artelt & Gräsel, 2009). Research on teacher judgement accuracy differentiates between relative and absolute judgement accuracy, respectively focusing on the relationship and the disparity between judgements and assessed cognitive abilities (Urhahne & Wijnia, 2021). This study examines relative judgement accuracy; thus, the magnitude of judgement inaccuracies cannot be considered. However, relative judgement accuracy does provide insights into teachers' ability to effectively differentiate between pupils with varying levels of understanding, which helps tailor instruction effectively (Thiede et al., 2019).

Within scholarly discourse, an interface exists between judgement accuracy and teacher expectations (Meissel et al., 2017). Both entail subjective evaluations. Nonetheless, while expectations usually entail anticipations about future accomplishments, judgements provide evaluations of pupils' current performance (Meissel et al., 2017). However, judgements serve as the underpinning for expectations (Meissel et al., 2017). This study focuses specifically on judgements, with explicit references made when prior literature addresses expectations.

### Teacher's and parent's judgements: accuracy and differences

Both teachers' and parents' judgements of pupils' cognitive abilities significantly impact their developmental opportunities (Benner et al., 2021). Teachers who underestimate pupils' achievement plan less challenging tasks, thus limiting learning (Rubie‐Davies et al., 2006), while overestimation can lead to insufficient guidance and overly complex tasks (Johnston et al., 2019). Underestimation also affects pupils' self‐perception, leading them to expect less success (Urhahne et al., 2011). This impacts their daily academic efforts, course choices, as well as their learning outcomes and aspirations (Mathew, 2017).

It is thus important that their judgements are accurate. Accurate alignment between teachers' and parents' judgements is thought to help create an optimal and stimulating learning environment for pupils (Glueck & Reschly, 2014). For instance, Peet et al. (1997) demonstrated that pupils whose mothers and teachers shared aligned perceptions regarding their competence, achieved higher grades compared to pupils whose mothers and teachers held less congruent views. Aligned judgements provide coherent rather than conflicting feedback (Peet et al., 1997), especially when positive as shown by Benner and Mistry (2007). They found that shared positive expectations regarding pupils finishing high school correlated with better test scores compared to shared negative expectations. Moreover, the impact was less significant when expectations did not align. Similar results were found by Rothenbusch et al. (2018), who examined the effect of teachers' and parents' ratings of gifted pupils' cognitive abilities on their German grades, controlling for assessed cognitive ability. However, causality remains unclear. This is supported by the longitudinal study of Mistry et al. (2008), which found that educational expectations of both mothers and teachers jointly influence and are influenced by pupils' school performance. Spera and Matto (2007) further theorized in their contextual‐congruence model that alignment fosters pupils' behavioural and social commitment in educational and familial environments.

This implies that it is beneficial that teachers and parents find common ground in their judgements (Rothenbusch et al., 2018). Teachers' and parents' judgements may differ, because they view pupils from different contexts and perspectives (Milic & Simeunovic, 2022; Rothenbusch et al., 2018). Teachers tend to focus on academic abilities and achievement (Jabůrek et al., 2022; Urhahne & Wijnia, 2021). Parents consider various contexts, such as home, social settings and special events, and they have known their children for longer (Sommer et al., 2008). However, parents have a smaller peer comparison group than teachers, making accurate judging harder (Milic & Simeunovic, 2022; Rothenbusch et al., 2018). Teachers, by contrast, judge pupils more frequently and professionally (Milic & Simeunovic, 2022).

Few studies have examined teacher and parent judgement of cognitive ability together, but research thus far has indicated moderate to high correlations between judgements and measured cognitive ability (Chamorro‐Premuzic et al., 2009; Sommer et al., 2008). Chamorro‐Premuzic et al. (2009) found teachers' judgements to be significantly more accurate than those of parents, who tended to overestimate pupils' cognitive abilities.

While teachers' and parents' judgements of pupils' cognitive abilities are reasonably accurate, other research indicates potential biases, particularly regarding SEN, gifted and low and middle SES pupils (e.g., Hurwitz et al., 2007; Wang et al., 2018). The following paragraphs will elaborate on this.

### Judgements of SEN pupils

In our study, SEN refers to pupils requiring special educational services due to a learning or behavioural need, in particular dyslexia, autism, ADHD, or ADD. SEN status has been shown to influence teachers' and parents' judgements of pupils' cognitive abilities (e.g., Banks et al., 2016; Wang et al., 2018). For instance, Hurwitz et al. (2007) showed that teachers' judgement of overall math performance and predicted math test scores were less accurate for SEN pupils, who were more likely to be underestimated. Cosgrove et al. (2014) found similar results for both teachers and parents, regarding reading and math. Additionally, Banks et al. (2016) and Whitley (2010) found lower expectations of future educational achievement for SEN pupils, even after accounting for actual cognitive ability.

The lower judgements of, and expectations for SEN pupils may stem from stigmatization or negative feelings, attitudes and beliefs surrounding their conditions (Hornstra et al., 2010; Jenkins & Demaray, 2016; Jussim & Harber, 2005; Zee et al., 2020). When stigmatization occurs, teachers and parents may rely on preconceived stereotypes, attitudes or believes, leading to lower expectations and differential treatment (Hornstra et al., 2010; Jussim & Harber, 2005). These biases are often implicit (Denessen et al., 2022). Moreover, teachers can experience less closeness and more conflict with SEN pupils, who often experience academic, behavioural, social, and emotional challenges, which may negatively influence judgements of their cognitive abilities (Zee et al., 2020).

### Judgements of gifted pupils

There is no consensus on the definition of giftedness (Türkman, 2020), though it is frequently viewed as encompassing advanced cognitive abilities, creativity, and strong intrinsic motivation (Altintas & Ilgün, 2015; Endepohls‐Ulpe & Ruf, 2005). In this study, parents indicated whether their child was gifted. Generally, gifted pupils show higher academic performance than average pupils. Teachers and parents find it easier to accurately judge high‐performers (Phillipson, 2010; Urhahne & Wijnia, 2021), due to their standing out compared to their peers (Coladarci, 1986). However, academic achievement is not a perfect indicator of cognitive abilities (Lavrijsen & Verschueren, 2020), as some gifted pupils underachieve (Siegle & Mc Coach, 2018). A meta‐analysis by Machts et al. (2016) found only a weak correlation between teachers' judgements of giftedness and measured cognitive abilities, while Rothenbusch et al. (2018) reported that both teachers' and parents' ratings were moderately accurate. This implies challenges in accurately judging the cognitive abilities of gifted pupils.

### Judgements of low‐to‐middle SES pupils

In the present study, highest family educational attainment is used as the indicator for SES. A lower SES is often associated with underestimation of cognitive abilities (e.g., Pinquart & Ebeling, 2020; Wang et al., 2018). Pupils from higher SES backgrounds tend to receive more favourable school track recommendations and have greater university attendance rates, even if their cognitive abilities are lower (De Boer et al., 2010; Paulus et al., 2021). Additionally, both parents and teachers have lower expectations regarding (future) educational attainment of pupils from low and middle SES families than high SES pupils, even when their academic performance is high (De Boer et al., 2010; Davis‐Kean, 2005; Van den Bergh et al., 2010; Wang et al., 2018;).

This underestimation might stem from stereotypes about lower SES pupils (Jussim & Harber, 2005). Moreover, parental involvement might play a role. Bakker et al. (2007) showed that low and middle SES parents perceived themselves as participating less in school activities compared to high SES parents. Teachers shared this perception and further believed that low and middle SES parents have less contact with teachers, less influence on school policy, and are less involved in supporting school activities at home. These perceptions may lead to teacher stereotypes about the parental involvement of all low and middle SES pupils (Bakker et al., 2007). The perception of reduced contact with low and middle SES parents might result in teachers providing less additional support for and having less insights about these students, potentially leading to inaccurate judgements about their abilities. Additionally, the perceived lower parental involvement can negatively impact pupils' academic achievement (Bakker et al., 2007), which might contribute to underestimation of these pupils' cognitive abilities. Last, low and middle SES parents spend less on socio‐cultural activities and materials for their children (Hao & Yeung, 2015). As a result, pupils from low‐to‐middle SES backgrounds have fewer opportunities to engage in enriching activities and access materials that stimulate their development by building skills, attitudes, behaviours, and social networks that are valued in the education system (Munir et al., 2023; Sirin, 2005). This might result in inaccurate judgements of their cognitive abilities.

### The present study

The present study investigates the relation between pupils' cognitive abilities and their teachers' and parents' judgements of these abilities. In addition, this study aims to investigate potential sources of systematic bias in these judgements caused by SEN, giftedness, and SES. As we theorize that these judgements are grounded in observations of the pupils' actual cognitive performance and not vice versa, we analysed how well assessed cognitive ability scores predict parent and teacher judgements of pupils' cognitive abilities.

To this end, the following research questions and hypotheses were addressed:

1. To what extent is there a relation between pupils' cognitive ability scores and teacher and parent judgements of pupils' cognitive abilities?
  1a. To what extent are cognitive ability scores predictive of teacher and parent judgements of pupils' cognitive abilities?
    (a) In line with findings from prior studies (e.g., Chamorro‐Premuzic et al., 2009; Urhahne & Wijnia, 2021), we hypothesized that both judgements have predictive value for pupils' measured cognitive abilities.
  1b. To what extent do teacher judgements of pupils' cognitive abilities differ from parent judgements?
    (b) We expected teachers' judgements to be lower than parents' (Chamorro‐Premuzic et al., 2009; Furnham & Valgeirsson, 2007)
  1c. To what extent does the predictive value of cognitive ability scores on judgements of pupils' cognitive abilities differ between teachers and parents?
    (c) We hypothesized that the predictive value of these judgements would be higher for teachers than for parents (Chamorro‐Premuzic et al., 2009).
2. Are there biases in teachers' and parents' judgements?

Do teachers and parents judge the cognitive abilities of (2a) SEN pupils lower than pupils without SEN, (2b) gifted pupils higher than not gifted pupils, (2c) low‐to‐middle SES pupils lower than high SES pupils, when correcting for their actual abilities? And does this bias differ in strength between parents and teachers?
(a) We expected both teachers' and parents' judgements to be lower for SEN pupils than for pupils without SEN (e.g., Banks et al., 2016; Hurwitz et al., 2007), (b) and higher for gifted pupils than for pupils who are not gifted (Baudson et al., 2016; Golle et al., 2023). (c) Last, we hypothesized that judgements of low‐to‐middle SES pupils were lower than high SES pupils' (e.g., De Boer et al., 2010; Van den Bergh et al., 2010; Wang et al., 2018). We will investigate the differences between teachers and parents regarding these potential biases.

The present study adds to the scientific literature for several reasons. First, unlike much of the existing research which typically focus on the judgements made by either teachers or, less frequently, parents alone, our study delves into both, enabling a direct comparison of their influences and disparities in judgement under identical conditions for the same group of pupils. Furthermore, our focus lies specifically on the evaluation of cognitive abilities, which form the foundation of individual potential. We examine separate cognitive abilities, recognizing the importance of addressing variations in strengths and weaknesses across these abilities. Moreover, as suggested in Urhahne and Wijnia's (2021) meta‐analyses, we looked at the influence of sociodemographic characteristics on judgements. Finally, we deem it important to replicate the studies of similar focus, in different cultural contexts, to expand the knowledge base.

## MATERIALS AND METHODS

The present study is part of a larger study on the validation of the COVAT‐3 for the Netherlands.

### Participants

A total of 1253 pupils received consent to participate. The total number of invited parents was not disclosed to the researchers, but based on the average class size in the Netherlands, a response rate of 66%–74% is estimated. As some children were absent at the day of testing, a total of 1073 primary school pupils from years 4 (37.6%), 5 (32.6%), and 6 (29.8%) from 77 classes in 16 schools, their teachers, and one of their parents was included in this study. One school was a school for full‐time gifted education (2.2% of the participants), the others were regular schools. In total, 48.7% of the participating pupils were girls, 50.9% boys, and .4% answered ‘different’ or ‘I do not want to say’. The pupils were between 6.57 and 14.38 years old (*M*_age_ = 10.79, SD_age_ = .96). Parents were asked if their child had a SEN. Among the pupils, 17.9% had a SEN (dyslexia, autism, ADHD, or ADD), 9.7% were gifted (with or without other SEN), and 72.5% had no SEN.

Parents were also asked to report their own and, if applicable, the other caregiver's highest completed educational level. The highest level of education of the caregivers indicated pupils' SES. In total, 25.7% of the pupils had a low‐to‐middle SES (i.e., elementary school, pre‐vocational secondary education, high school or secondary vocational education) and 74.3% of pupils had a high SES (i.e., higher education). The parent who completed the questionnaire was predominantly the mother (81.1%; father 18.4%; .5% other caregiver).

### Procedure

The study was approved by the ethics committee of Fontys University of Applied Sciences. The participating schools were part of a professional learning community dedicated to collaborative research on educational practices for gifted pupils. Teachers within this community informed their colleagues about the research and invited them to participate voluntarily. Parents received information about the study through an online letter from their child's teacher and provided active consent for both themselves and their child(ren). They completed an online questionnaire at their chosen time and place. Thereafter, a trained researcher or graduate student administered a cognitive ability test at the schools during school hours. The test was administered in a classroom, in silence, with all participating pupils from the same class. The test took about 75 min (no breaks). The pupils completed the test independently, within the given time limit per test item. Teachers were asked to fill out an online questionnaire during the test administration.

### Instruments

#### Cognitive abilities

Pupils' cognitive abilities were measured with the psychodiagnostic instrument COVAT‐3, a digital cognitive ability test developed in Flanders, the Dutch‐speaking region of Belgium (Magez et al., in preparation). The linguistic variation between Flanders and the Netherlands is similar to the differences between American and British English. COVAT stands for Cognitieve VaardighedenTest (Cognitive Ability Test), 3rd version. The COVAT‐3 is in development, building upon the foundation laid by the COVAT‐CHC (Magez, De Cleen, et al., 2015; Magez, Tierens, et al., 2015), a validated (Magez & Bos, 2016; Tierens, 2017) and reliable test (Decaluwé et al., 2017; Tierens & Magez, 2016) that has received the highest quality rating (A+) from the Belgian Test Committee (Belgian Federation of Psychologists, 2021). The COVAT‐CHC has been standardized for children aged 9 years and 6 months up to 13 years and 11 months in Belgium and for primary school and secondary school classes in Belgium (Tierens & Magez, 2016). Improvements of the COVAT‐3 compared to earlier versions are the digital format, the adaptive format, and as a result also a shortened total test time.

The test is based on the CHC‐model of intelligence and measures six broad cognitive abilities: reasoning, comprehension, visual processing, STM, LTM and processing speed. Each cognitive ability was measured with one subtest, except for comprehension, which was measured with two subtests. The subtests of STM, LTM and processing speed, existed in a visual and a verbal format. The administration of both formats would take too long. To obtain information about both formats for the validation of the COVAT‐3, pupils were allocated to either the verbal tasks or the visual tasks: the first half of each class (based on last name) received the verbal tasks and the second half the visual tasks. All other tasks were completed by the entire group.

Reliability analyses indicated satisfactory to good internal consistency for each subtest: reasoning α = .79, comprehension α = .85 and α = .72, visual processing α = .88, verbal version of STM α = .83 and LTM α = .81, visual version of STM α = .86, and LTM α = .88. For processing speed, reliability could not be calculated due to its nature as a speed task.

Standardized norms for the COVAT‐3 are currently not available for the Netherlands. Therefore, the raw total scores were used. As the test scales differed across instruments, z‐scores were taken for each sub‐test. The z‐scores of the two subtests for comprehension were averaged to create a composite comprehension score. To correct for age differences, age‐residualized scores were taken. To do this, the sub‐test scores were regressed on age and the difference between the obtained and age‐predicted scores were used as an age‐adjusted score. Together, this procedure enabled comparison of scores across the different abilities and for pupils of different ages.

#### Teachers' and parents' judgements of pupils' cognitive abilities

The judgements of pupils' cognitive abilities by their parents and teachers were measured with six statements, each corresponding to a cognitive ability of the COVAT‐3 (see Table 1). These statements were formulated based on the definitions as outlined by Magez, De Cleen, et al. (2015) and Magez, Tierens, et al. (2015). Each statement indicates a behaviour exhibited by pupils that serves as an indicator of a cognitive ability, observable by teachers and parents. This method of assessing respondents' judgements of pupils' cognitive abilities has been used in similar studies (e.g., Baudson et al., 2016; Gnas et al., 2022). Respondents were asked to place a marker on a continuum ranging from ‘totally disagree’ to ‘totally agree’. These answers were converted into a continuous score from 0 to 100.

**TABLE 1:** Statements for parents and teachers concerning pupils' cognitive abilities.

Cognitive ability	Statement (translated)
Fluid reasoning	This pupil/my child is good at solving problems in new situations
Comprehension knowledge	This pupil/my child has a lot of general knowledge
Visual processing	This pupil/my child is good at solving puzzles with pictures or figures
Short‐term memory	This pupil/my child can remember information, such as a complicated address, for a few minutes
Long‐term storage and retrieval	This pupil/my child remembers a lot, including things that happened a long time ago
Processing speed	This pupil/my child can complete simple assignments very quickly

### Data analysis

#### Missing values

Missing data were observed. Specifically, 170 pupils (13.6%) lacked teacher judgements, 22 pupils (1.8%) lacked parental judgements, with 8 pupils (.6%) missing both. Parental background information was absent for 8 pupils (.6%). Additionally, 152 pupils (12.1%) did not complete the COVAT‐3. Several factors contributed to this missing data. Data collection occurred during the COVID‐19 pandemic, which likely reduced pupil participation due to illness. Teachers did not always provide judgements for absent pupils. Technical issues at one school resulted in missing data for 84 pupils and their teachers. Furthermore, there were 23 missing values (1.8%) for SES (including ‘do not want to say’ answers) and 31 (2.5%) for SEN.

Little's MCAR test indicated that the missingness on these sources was unrelated to each other, as well as to age, gender, school and class (χ^2^(9) = 12, *p* = .219), suggesting that the missing values were missing at random. Pupils lacking both parental and teacher judgements, not completing any COVAT‐3 subtest, or missing all parental background information were excluded from the analysis. Missing values on SEN and SES were handled using Multiple Imputation, a standard procedure in IBM SPSS Statistics 29. The Fully Conditional Specification (FCS) method was applied with 10 iterations. Five imputed datasets were generated, and the pooled results from these datasets were used for subsequent analyses.

#### Outliers

Outliers and influential cases were identified and addressed through a two‐step process. First, univariate outliers were detected by examining *z*‐scores, with values >|3| considered outliers. Winsorizing was applied to minimize their influence. Second, Mahalanobis Distance was calculated to detect multivariate outliers. Several multivariate outliers were identified. Analyses were conducted both with and without these multivariate outliers to assess their impact. The presence of these multivariate outliers did not change the results. Therefore, the results from the full sample are reported.

#### Main analyses

The data had a nested structure, with judgements and cognitive ability scores being nested in pupils and pupils in classes. Therefore, multilevel analyses were conducted. Separate multilevel analyses were run for each cognitive ability, thus six analyses in total.

Judgements of pupils' cognitive abilities were the dependent variables in the analyses. We estimated random‐intercept models to examine the intra‐class correlations (ICC). Model 1 contains the COVAT‐3 scores, the informant of the predictions (reference category = parent), SEN and gifted (reference category = other pupils) and SES (reference category = high). The analyses with STM, LTM and decision speed as depended variable also included the version of the COVAT‐3 (reference category = verbal version). The effect of the COVAT‐3 score answers whether pupils' assessed cognitive abilities had predictive value for teachers' and parents' judgements. Informant indicates if teachers' and parents' judgements regarding the cognitive abilities of pupils differed in mean level, when correcting for pupils' assessed cognitive abilities. The effects of pupils' SEN and gifted status answer the questions if teachers' and parents' judgements differed in mean level for gifted pupils and SEN pupils compared to the ‘other’ group. The effect of SES shows whether these judgements differed for low‐to‐middle SES pupils compared to high SES. In Model 2, the interaction terms of informant × COVAT‐3, informant × SEN, informant × gifted, and informant × SES were added. The added interaction terms answer the question whether the found effects of COVAT‐3, SEN and SES in Model 1 differed between teachers and parents. The explained variance by the model was assessed using marginal and conditional *R* ^2^. Marginal *R* ^2^ measures the variance explained by fixed effects, while conditional *R* ^2^ also includes the random effects (Nakagawa & Schielzeth, 2013). Effect sizes (ES) were computed based on Cohen's *f* ^2^ (Cohen, 1988; Selya et al., 2012). Effect sizes of *f* ^2^ ≥ .02, *f* ^2^ ≥ .15, and *f* ^2^ ≥ .35 represent small, medium, and large effect sizes, respectively (Cohen, 1988).

Only pupils who had completed a cognitive ability task were included in the analysis of that cognitive ability. A Bonferroni correction for multiple testing (6 analyses; α = .05/6 = .008) was used, as the analyses were performed separately for each cognitive ability. The assumptions were checked for all analyses. The assumptions of absence of multicollinearity and linearity were met. The assumption of normality of residuals for the dependent variables was not perfectly met. We used the restricted maximum likelihood to ensure robustness of the analyses despite the small deviation from normality.

### Pre‐registration and deviations from the pre‐registration

The design of the study and the analysis plan of the study were pre‐registered via the Open Science Framework (https://doi.org/10.17605/OSF.IO/WDNM5). The pre‐registration occurred after data collection, but before data review and analysis. There were deviations from the pre‐registration: We pre‐registered to divide the SES variable into three categories (low‐middle‐high) but due to the small low SES sample (*N* = 34), the categories low and middle SES were merged. Subsequently, we pre‐registered two sets of analyses. However, we realized that only one set of analyses was needed to answer all questions, by adding an interaction term between informant and COVAT‐3. Furthermore, Models were adjusted, adding the variable COVAT‐3 already in Model 1 to control for pupils' assessed cognitive abilities and the variables SEN, gifted and SES already in Model 1 to show the overall mean level differences between these categories. Furthermore, Model 0 was omitted considering the limited information it provides. Finally, we only pre‐registered to take pupils' class as nested variable. But because each pupil was rated by both a teacher and a parent, we also modelled pupil as a level.

## RESULTS

### Descriptive statistics and correlations

Table 2 shows the descriptive statistics of teachers' and parents' judgements and pupils' COVAT‐3 scores (prior to the standardization procedure). Table 3 shows the correlations between judgements and COVAT‐3 scores. The correlations indicated that parents' and teachers' judgements were weakly to strongly correlated. Parents' judgements and COVAT‐3 scores were overall weakly correlated. Teachers' judgement had weak to moderate correlations with COVAT‐3 scores.

**TABLE 2:** Descriptive statistics of teachers' and parents' judgements of pupils' cognitive abilities and pupils' raw COVAT‐3 scores.

	Teacher				Parent				Pupils' raw COVAT‐3 scores			
	Min.	Max.	M	SD	Min.	Max.	M	SD	Min.	Max.	M	SD
Fluid reasoning	0	100	61.2	24.9	0	100	63.9	22.9	8	41	24.7	5.2
Comprehension knowledge	0	100	66.3	24.9	0	100	71.6	21.2	0	60	37.1	8.2
Visual processing	0	100	65.2	25.5	0	100	73.6	21.8	0	67	24.1	10.8
*Verbal format*												
Short‐term memory	0	100	65.8	25.6	0	100	73.5	22.9	0	36	9.6	5.9
Long‐term storage and retrieval	6	100	70.0	21.9	0	100	81.7	18.7	2	28	9.8	5.6
Processing speed	0	100	68.8	26.6	0	100	76.2	22.3	16	287	78.6	29.8
*Visual format*												
Short‐term memory									0	28	12.1	5.3
Long‐term storage and retrieval									1	28	13.4	5.6
Processing speed									0	90	39.9	14.1

**TABLE 3:** Correlations between teachers' and parents' judgements and pupils' assessed cognitive ability scores.

Variable	1	2	3	4	5	6	7	8	9	10	11	12	13	14	15	16	17
*Parent*																	
1. Reasoning																	
2. Comprehension	.46***																
3. Visual processing	.43***	.51***															
4. STM	.41***	.56***	.52***														
5. LTM	.27***	.46***	.41***	.52***													
6. Processing speed	.43***	.47***	.54***	.57***	.36***												
*Teacher*																	
7. Reasoning	.30***	.31***	.28***	.30***	.14***	.35***											
8. Comprehension	.22***	.42***	.27***	.34***	.20***	.31***	.73***										
9. Visual processing	.22***	.37***	.27***	.36***	.17***	.35***	.69***	.81***									
10. STM	.25***	.37***	.29***	.38***	.20***	.37***	.72***	.79***	.79***								
11. LTM	.20***	.33***	.23***	.33***	.21***	.29***	.66***	.77***	.75***	.77***							
12. Processing speed	.22***	.33***	.25***	.32***	.15***	.37***	.70***	.72***	.80***	.77***	.71***						
*COVAT‐3 scores*																	
13. Reasoning	.15***	.23***	.23***	.20***	.08*	.24***	.28***	.32***	.35***	.34***	.26***	.33***					
14. Comprehension	.19***	.39***	.25***	.32***	.22***	.26***	.37***	.50***	.44***	.45***	.41***	.41***	.40***				
15. Visual processing	.04	.09*	.16***	.14***	.13***	.12***	.15***	.21***	.20***	.18***	.18***	.19***	.30***	.34***			
16. STM	.11***	.23***	.20***	.20***	.14***	.13***	.21***	.27***	.26***	.23***	.24***	.24***	.25***	.38***	.28***		
17. LTM	.11***	.25***	.23***	.21***	.15***	.16***	.24***	.29***	.28***	.29***	.28***	.29***	.26***	.40***	.26***	.77***	
18. Processing speed	.16*	.17***	.20***	.17***	.15***	.22***	.26***	.25***	.25***	.23***	.23***	.25***	.28***	.31***	.27***	.27***	.28***

* *p* < .05.

*** *p* < .001.

### Intra‐class correlations

The ICCs are summarized in Table 4. In the different analyses, between 2% and 5% of the variance in teachers' and parents' judgements was located at the class level, suggesting that there were limited differences in judgements between classes. While this level of variance seems small, even small ICCs could distort the results (Cohen et al., 2003). A larger proportion of variance was situated at the pupil level, between 6% and 37%. This indicates meaningful differences in judgements of informants between pupils and differences in judgements between informants, justifying the use of multilevel analyses.

**TABLE 4:** Intra‐class correlations.

Cognitive ability	ICC class level	ICC pupil level
Fluid reasoning	.03	.26
Comprehension	.03	.37
Visual processing	.03	.21
Short‐term memory	.02	.33
Long‐term storage and retrieval	.05	.06
Processing speed	.02	.31

### Explained variance

Tables 5 and 6 show the results of the multilevel analyses, including the marginal and conditional *R* ^2^. The results show that the pupil characteristics accounted for between 10% and 25% of the variance in judgements. This suggests that other pupil characteristics beyond the factors in this study also have a significant impact on the judgements. When accounting for both the class‐level and the pupil‐level variability, the model explained between 27% and 40% of the variation in judgements. This indicates that the differences among classes and, particularly, among individual pupils and informants contribute substantially to the overall explanation of the judgements made by teachers and parents. However, a substantial portion of the variance remains unexplained by the included factors, suggesting that other unmeasured variables might play a significant role in these judgements. Overall, introducing the interaction terms did not result in a substantial change in the explained variance.

**TABLE 5:** Predicting parents' and teachers' judgements of pupils' fluid reasoning, comprehension knowledge and visual processing.

Fixed effects	Fluid reasoning *N* = 1081				Comprehension knowledge *N* = 1089				Visual processing *N* = 1057			
	Model 1		Model 2		Model 1		Model 2		Model 1		Model 2	
	*B*	SE	*b*	SE	*b*	SE	*b*	SE	*b*	SE	*b*	SE
Intercept	67.12***	.99	66.22***	1.05	72.63***	.87	71.29***	.90	75.63***	.96	73.99***	1.03
COVAT‐3 score	4.24***	.57	2.86***	.69	8.88***	.54	7.23***	.67	3.55***	.56	3.25***	.71
Informant^a^	−2.63**	.88	−.78	1.52	−5.28***	.77	−2.48	1.00	−8.07***	.90	−4.67***	1.15
SEN	−10.80***	1.45	−9.22***	1.78	−3.2	1.34	−2.10	1.67	−9.20***	1.46	−7.28***	1.85
Gifted	1.61	2.02	−.10	2.37	10.49***	1.81	10.96***	2.24	8.76***	2.01	5.85	2.52
Low SES	−6.48***	1.28	−3.50	1.56	−5.75***	1.16	−1.58	1.45	−4.94***	1.30	1.22	1.63
Informant × COVAT‐3			2.85**	.88			3.44***	.83			.63	.89
Informant × SEN			−3.29	2.25			−2.51	2.07			−4.00	2.37
Informant × Gifted			3.40	3.16			−1.15	2.67			5.78	3.06
Informant × low SES			−6.02**	1.99			−8.47***	1.78			−12.48***	2.03
*Random effects*												
Class variance	22.00**		21.94**		11.98		11.87		16.44		16.57	
Pupil variance	93.75***		99.67***		9187.9***		96.40***		93.26***		103.3***	
*R* ^2^ marginal	.10		.11		.25		.26		.28		.13	
*R* ^2^ conditional	.30		.32		.43		.46		.34		.33	
−2 Log likelihood	18,999		18,955		18,655		18,585		18,588		18,520	

^a^ Parent = 0, Teacher = 1.

** *p* < .008.

*** *p* < .001.

**TABLE 6:** Predicting parents' and teachers' judgements of pupils' short‐term memory, long‐term storage and retrieval and processing speed.

Fixed effects	Short‐term memory *N* = 1073				Long‐term storage and retrieval *N* = 958				Processing speed *N* = 1051			
	Model 1		Model 2		Model 1		Model 2		Model 1		Model 2	
	*b*	SE	*b*	SE	*b*	SE	*b*	SE	*b*	SE	*b*	SE
Intercept	77.18***	1.11	76.26***	1.16	83.45***	1.00	82.28***	1.05	79.85***	1.17	78.54***	1.22
COVAT‐3 score	4.20***	.57	3.68***	.70	3.40***	.49	2.34***	.64	4.95***	.61	4.30***	.75
Version^a^	.27	1.10	.25	1.10	−.93	.93	−.43	.93	−.19	1.14	−.18	1.14
Informant^b^	−7.65***	.84	−5.70***	1.10	−11.44***	.84	−8.97***	1.08	−7.41***	.87	−4.71***	1.13
SEN	−14.76***	1.55	−13.87***	1.88	−7.22***	1.32	−5.38**	1.70	−13.21***	1.56	−11.70***	1.94
Gifted	7.63***	2.05	6.78**	2.52	8.69***	1.70	6.59**	2.19	−2.41	2.12	2.14	2.60
Low SES	−7.57***	1.33	−4.21	1.63	−3.53**	1.13	−.76	1.48	−5.19***	1.37	−1.09	1.68
Informant × COVAT‐3			1.12	.86			2.23	.85			−1.35	.91
Informant × SEN			−1.86	2.29			−3.78	2.27			−3.14	2.34
Informant × Gifted			1.64	2.95			4.16	2.79			.48	3.00
Informant × Low SES			−6.84***	1.95			−8.62***	1.92			−8.30***	2.00
Random effects												
Class variance	16.77		16.77		15.93**		15.63**		19.84		19.84	
Pupil variance	130.64***		133.41***		34.43**		41.46***		135.93***		140.00***	
*R* ^2^ marginal	.16		.16		.16		.18		.13		.14	
*R* ^2^ conditional	.40		.41		.27		.31		.38		.40	
−2 Log likelihood	18,796		18,764		16,261		16,205		18,523		18,484	

^a^ Verbal format = 0, Visual format = 1.

^b^ Parent = 0, Teacher = 1.

** *p* < .008.

*** *p* < .001.

### Relation between cognitive ability scores and judgements

Tables 5 and 6 show the results of the multilevel analyses.

#### Overall predictive value

Regarding research question 1a, Model 1 reports the findings for the effect of COVAT‐3 scores on judgements. For all cognitive abilities, COVAT‐3 scores were significant predictive for judgements. However, the effect sizes were small for most cognitive abilities (*f* ^2^ = .02 to .04), except for comprehension (*f* ^2^ = .16).

#### Differences in mean level

Answering research question 1b, the effect of informant in Model 1 indicated that for all cognitive abilities, teachers' judgements were significantly lower than parents' judgements. This difference was greatest for pupils' LTM (*b* = −11.44, *p* < .001) and smallest for reasoning (*b* = −2.63, *p* = .003). Effect sizes were small (*f* ^2^ = .02 to .09), and negligibly small for reasoning.

#### Differences in predictive value

In Model 2, the interaction term informant × COVAT‐3 addresses research question 1c. Indicating that there was no difference in the predictive value of COVAT‐3 scores between teachers and parents across four out of the six cognitive abilities. For reasoning and comprehension, the interaction was significant. A simple slopes analysis (Appendix 1) revealed that the predictive value of respectively reasoning and comprehension for teachers was *b* = 5.81, *p* < .001 and 10.87, *p* < .001 compared to *b* = 2.92, *p* < .001 and *b* = 7.07, *p* < .001 for parents. The 95% confidence intervals did not overlap, signifying that teachers' judgements held significantly more predictive value than those of parents. However, these effects were negligible in size.

### Bias in judgements

#### SEN

Corrected for pupils' COVAT‐3 scores, five out of six cognitive abilities of SEN pupils were judged as lower than the cognitive abilities of other pupils. The most substantial disparities were evident in STM (*b* = **−**14.76, *p* < .001) and processing speed (*b* = **−**13.21, *p* < .001). SEN pupils' comprehension was not judged significantly differently. The effect sizes were small (*f* ^2^ = .02 to .04), and negligible for comprehension. The interaction term informant × SEN in Model 2 was not significant, indicating that the bias in judgements of the cognitive abilities of SEN pupils did not differ between teachers and parents.

#### Giftedness

Correcting for pupils' COVAT‐3 scores, parents' and teachers' judgements of gifted pupils' cognitive abilities did differ significantly from those of other pupils for four out of the six cognitive abilities. Their judgements of gifted pupils' comprehension, visual processing, STM, and LTM were higher than for other pupils. The effect sizes were small (*f* ^2^ = .02), and negligible for LTM. These biases in judgements did not differ between teachers and parents.

#### SES

Finally, judgements of low‐to‐middle SES pupils' cognitive abilities were lower compared to those of high SES pupils, correcting for assessed abilities. Effect sizes of reasoning, comprehension and STM were small (*f* ^2^ = .02) the others were negligibly small.

The significant negative interaction term informant*SES indicates a difference between teachers and parents. Simple slopes analyses (Appendix 2) revealed that only teachers, not parents, had a bias in their judgements of low‐to‐middle SES pupils' cognitive abilities compared to high SES pupils. However, all effect sizes were negligible.

## DISCUSSION

Every pupil has strengths and weaknesses in certain cognitive abilities. The ability of both teachers and parents to judge pupils' potential accurately and without bias is essential. It ensures that pupils receive learning environments supportive to their intellectual growth and educational success (Fischbach et al., 2013; Gentrup et al., 2020). Therefore, the present study aimed to gain insight into the relation between pupils' cognitive abilities and their teachers' and parents' judgements of these abilities. In addition, this study aimed to investigate potential sources of systematic bias in these judgements caused by SEN, giftedness, and SES. A multilevel approach was used.

### Relation between cognitive ability scores and judgements

Our first research question concerned the relation between pupils' cognitive ability scores and teacher and parent judgements of pupils' cognitive abilities. Consistent with our hypothesis (H1a), COVAT‐3 scores were predictive for teachers' and parents' judgements, suggesting their ability to judge pupils' cognitive abilities to some degree. However, the strength of the predictive value was modest. Regarding H1b, which anticipated differences in judgements between teachers and parents, we observed that teachers tended to rate pupils lower compared to parents. Again, the practical significance of these differences was limited. Last, contrary to our expectation (H1c), these differences did mostly not significantly affect the predictive value. Only for reasoning and comprehension, COVAT‐3 scores seemed more predictive of teachers' judgements than parents', however effect sizes were negligibly small. Prior research has identified variations in judgement accuracy between fathers and mothers (Chamorro‐Premuzic et al., 2009), noting that judgements of mothers were more aligned with teachers' judgements than fathers' judgements. Given that most parents in our study were mothers (81%), this could explain the absence of significant differences. Furthermore, overall, the correlations between judgements and COVAT‐3 scores were weak to moderate, suggesting that the statements used may not accurately represent the concepts, or neither teachers nor parents in our sample possess strong abilities in judging pupils' cognitive abilities (Waschbusch et al., 2000).

Thus, answering research question 1, it can be concluded that both teachers and parents exhibited limited proficiency in assessing pupils' cognitive abilities, and these capabilities were largely similar. This parity is crucial, as the alignment of positive assessments between teachers and parents has been argued to foster an optimal and stimulating learning environment for pupils (Glueck & Reschly, 2014). This research underscores the necessity for further investigation into the absolute accuracy of these judgements, examining whether they accurately reflect pupils' abilities or if there is a tendency to either overestimate or underestimate them.

### Bias in judgements

#### SEN

Our second question concerned possible biases in teacher and parent judgements, concerning SEN, giftedness and SES. As expected (H2a), SEN pupils' cognitive abilities, except for comprehension, were judged as lower than other pupils' cognitive abilities, controlled for assessed cognitive abilities. This bias did not differ between teachers and parents. While this finding is consistent with prior research (e.g., Cosgrove et al., 2014; Hurwitz et al., 2007), the magnitude of these biases was limited in our study. Nonetheless, this finding remains concerning as such biases, despite their modest size, may still impact the opportunities and support provided to SEN pupils both in the classroom and at home which may inhibit pupils' development and achievements (e.g., Rubie‐Davies et al., 2006). SEN pupils may be vulnerable to group stigmatization (Hornstra et al., 2010) or experience less closeness and more conflict with teachers, which might negatively influence judgements of these pupils' cognitive abilities (Jenkins & Demaray, 2016; Zee et al., 2020).

#### Giftedness

We hypothesized (H2b) higher judgements for gifted pupils than for other pupils. We found that most cognitive abilities of gifted pupils were judged higher than other pupils' cognitive abilities, even after accounting for assessed cognitive abilities. Judgements of fluid reasoning and processing speed did not differ between gifted pupils and other pupils, controlled for assessed cognitive abilities. There were no differences in this bias between teachers and parents. Again, the magnitude of this bias was limited. Nonetheless, this implies that gifted pupils are at risk of biased judgements. Such bias could affect the expectations placed on gifted pupils and the support they receive, such as being expected to work independently without guidance and instruction or receiving too challenging materials (Johnston et al., 2019; Rothenbusch, 2016). It is crucial for teachers and parents to hold judgements that accurately reflect gifted pupils' abilities, as significant overestimation could result in pressure and underestimation in boredom (Johnston et al., 2019). Therefore, future research should consider absolute accuracy.

#### SES

We hypothesized (H2c) that low‐to‐middle SES pupils' cognitive abilities would be judged lower than high SES pupils' cognitive abilities, even after accounting for assessed cognitive abilities. This bias was found for all cognitive abilities. However, the impact of the biases for reasoning, comprehension and STM was small and negligible for the other abilities. The SES bias was exclusively found for teachers, although its impact was negligible. Low‐to‐middle SES pupils might face stigmatization through parental involvement and having less access to enriched activities and materials, factors that may influence teachers' judgements (Bakker et al., 2007; Hao & Yeung, 2015; Sirin, 2005). Conversely, parents judge their children's cognitive abilities based on their own cultural norms and values, often without comparison with other children (Milic & Simeunovic, 2022). The observed bias, although small of impact, is concerning as it directly impacts the daily educational opportunities pupils receive (e.g., Rubie‐Davies et al., 2006). Moreover, given that pupils cannot alter their parents' level of education, this bias exacerbates social and structural injustices (Baudson et al., 2016). Further research is needed to explore the mediators of this bias.

### Factors influencing judgements

Interestingly, a substantial portion of the variance in teachers' and parents' judgements of pupils' cognitive abilities remained unexplained by the factors included in our study. This suggests that teachers and parents likely consider additional factors when assessing pupils' cognitive abilities. For instance, academic achievement may also play a critical role (Deunk et al., 2018). Alternatively, it is possible that teachers and parents do not have a clear understanding of pupils' cognitive abilities and have (partly) relied on guesswork.

### Limitations

The present study added to the scientific literature on judgements by directly comparing teachers' and parents' judgements of pupils' individual cognitive abilities using multilevel analyses. Pupils' SEN, giftedness and SES were highlighted. Some issues limited the present study.

First, reliance on parent reports for SEN/giftedness measures may have affected accuracy, as it is unclear whether teachers were aware of or agreed with these labels. Potential parental bias may therefore affect the strength of the relationship between parent judgements and pupils' abilities compared to teacher judgements. Ideally, a more comprehensive approach would involve formal diagnoses of SEN or giftedness, assessing diagnostic test outcomes and teacher judgements. However, studies show that the diagnostic accuracy rate of parents and teachers for diagnosing children with ADHD is similar and around 90% (Bied et al., 2017; Tahıllıoğlu et al., 2021). For ASD, mothers accurately identified 64% of children with ASD, while teachers identified 25% (Mayes & Lockridge, 2018). Thus, parental reports for pupils' SEN seem to be reasonably reliable as a measure to some degree. Additionally, concerning giftedness, the gifted pupils in our sample scored significantly higher on the COVAT than the other pupils. However, among the 2.5% highest‐scoring pupils, there were also pupils who were not labelled as gifted by their parents. Indicating that we might have missed some gifted pupils. Furthermore, relying on diagnostic test outcomes also has downsides, as students who were not considered for testing due to a lack of experienced problems may be missed.

Second, the sample was biased with 72.8% of pupils from high SES backgrounds, compared to the participating schools regional rate of 37%–47% (Compendium voor de Leefomgeving, 2023). Highly educated parents might have been more likely to take part in the study. This discrepancy affects the generalizability of the study's findings. Additionally, the low percentage of low SES pupils led to merging the low and middle SES pupils, where the middle SES category predominates. By combining these groups, the unique judgements of both groups may be overlooked. To address these limitations, future studies should aim to include a sufficiently large and representative sample of pupils from each SES category.

Furthermore, a limitation is the frequent use of the maximum score of 100 by both teachers and parents when judging pupils' cognitive abilities. Since the initial location of the marker was on the left side of the line, giving this highest score was done consciously. Possibly teachers and parents did not fully understand the relative nature of the statements in the questionnaire and how their responses should reflect a relative judgement rather than an absolute one. For future research, this could be stimulated by informing the respondents about the relative nature by adding ‘compared to peers’.

Fourth, parents' and teachers' judgements were measured using single‐items. While these measures were selected for their practicality and to reduce respondent burden, they have limitations in terms of validation and reliability. Single‐item measures may not fully capture the complexity and nuances of the cognitive abilities being assessed (Allen et al., 2022).

Fifth, the COVAT‐3 is still in development, and its reliability and validity are not yet fully established. However, it builds upon the COVAT‐CHC, which has been validated (Magez & Bos, 2016; Tierens, 2017), demonstrated reliability (Decaluwé et al., 2017; Tierens & Magez, 2016), and received a high‐quality rating from the Belgian Federation of Psychologists (2012). Dutch norm scores for the COVAT‐3 were not yet available, therefore raw scores were used, necessitating cautious interpretation. Additionally, the test's length and classroom administration may have affected pupils motivation and concentration, potentially impacting score reliability (Duckworth et al., 2011). Future research should consider multiple assessment points and designs as vignettes, to address these issues and explore potential biases in cognitive ability testing (Neisser et al., 1996).

Finally, the effect sizes observed in this study were small, indicating a limited impact. However, even small effects can accumulate over time to exert a significant influence (Abelson, 1985; Rosenthal & Rubin, 1994). This consideration is particularly important considering the biases identified and their potential long‐term impact on pupils.

## CONCLUSION AND IMPLICATION

The present study offered insights into teachers' and parents' judgements of pupils' cognitive abilities. Accurate judgements are crucial for pupils' educational success; therefore, they should be bias free. Both teachers and parents demonstrated similar, but limited, accuracy in their judgements. Biases were observed in their judgements of SEN, gifted, and low‐to‐middle SES pupils' cognitive abilities, although it is unclear whether these were over‐ or underestimated given the study's focus on relative accuracy. However, this study offers reason to delve further into these biases as they influence the learning environment that these pupils are offered.

Accurately assessing pupils' cognitive abilities, particularly for SEN and low‐to‐middle SES pupils, presents challenges for both teachers and parents. To improve accuracy, both parties must consider multiple perspectives, acquire relevant knowledge, and confront stereotypes and misconceptions. Encouraging discussions between teachers and parents, incorporating examples of cognitive behaviour from both contexts, can enrich perceptions and foster alignment. These efforts are crucial for creating a more equitable and effective educational environment for all pupils.

## AUTHOR CONTRIBUTIONS

**Kim Smeets:** Conceptualization; methodology; formal analysis; writing – original draft; investigation. **Ellen Rohaan:** Conceptualization; writing – review and editing; supervision. **Sanne van der Ven:** Conceptualization; methodology; supervision; writing – review and editing. **Anouke Bakx:** Conceptualization; funding acquisition; data curation; supervision; writing – review and editing.

## CONFLICT OF INTEREST STATEMENT

The authors declare that there is no conflict of interest.

## Data Availability

The research data are not shared due to privacy or ethical restrictions.

## APPENDIX 1

Simple slopes analysis for differences in predictive value of judgements of pupils' cognitive abilities between teachers and parents.

**Table:**

	Reasoning				Comprehension			
			95% confidence interval				95% confidence interval	
	*b*	SE	Lower bound	Upper bound	*b*	SE	Lower bound	Upper bound
Teacher								
Intercept	65.59***	1.40			69.24***	1.27		
COVAT‐3 score	5.81***	.71	4.41	7.21	10.87***	.69	9.51	12.22
Parent								
Intercept	66.36***	.89			71.12***	.78		
COVAT‐3 score	2.92***	.70	1.55	4.30	7.07***	.65	5.81	8.34

*Note*: Included but not shown fixed effects are COVAT‐3 score, test version, SEN and Gifted; random effects class and pupils.

*** *p* < .001.

## APPENDIX 2

Simple slopes analysis for differences in judgements of pupils' cognitive abilities between teachers and parents.

**Table:**

	Reasoning		Comprehension		Visual processing		STM		LTM		Processing speed	
	*b*	SE	*b*	SE	*b*	SE	*b*	SE	*b*	SE	*b*	SE
Teacher												
Intercept	65.59***	1.40	69.24***	1.27	69.31***	1.36	70.19***	1.52	73.60***	1.44	73.96***	1.74
Low SES	−10.58***	1.62	−11.08***	1.49	−12.11***	1.96	−12.11***	1.70	−8.73***	1.51	−10.40***	1.77
Parent												
Intercept	66.36***	.89	71.12***	.78	73.91***	.92	76.77***	1.13	82.22***	.99	78.36***	1.13
Low SES	−3.13	1.59	−1.05	1.39	1.70	1.56	−3.69	1.56	1.39	1.41	−.66	1.56

*Note*: Included but not shown fixed effects are COVAT‐3 score, test version, SEN and Gifted; random effects class and pupils.

*** *p* < .001.
